# Assessing the impact of COVID-19 on acute leukemia patients: a comparative analysis of hematological and biochemical parameters

**DOI:** 10.1186/s12879-024-09485-9

**Published:** 2024-06-11

**Authors:** Abdulaziz M. Almuqrin, Badi A. Alotaibi, Jehad A. Aldali, Abdulrahman Alshalani, Hamood AlSudais, Hamzah J. Aldali

**Affiliations:** 1 Chair of Medical and Molecular Genetics Research, Department of Clinical Laboratory Sciences, College of Applied Medical Sciences, King Saud University, Riyadh, 12372 Saudi Arabia; 2 Department of Clinical Laboratory Sciences, College of Applied Medical Sciences, King Saud Bin Abdulaziz University for Health Sciences, Riyadh, 11481 Saudi Arabia; 3 King Abdullah International Medical Research Center, Riyadh, Saudi Arabia; 4 Department of Pathology, College of Medicine, Imam Mohammad Ibn Saud Islamic University (IMSIU), Riyadh, 13317 Saudi Arabia; 5 Cellular and Molecular Medicine, College of Biomedical Science, University of Bristol, Bristol, BS8 1QU UK

**Keywords:** COVID-19, Acute leukemia, Outcomes, Mortality, Survival analysis

## Abstract

**Background:**

The impact of COVID-19 infection on the blood system remains to be investigated, especially with those encountering hematological malignancies. It was found that a high proportion of cancer patients are at an elevated risk of encountering COVID-19 infection. Leukemic patients are often suppressed and immunocompromised, which would impact the pathology following COVID-19 infection. Therefore, this research aims to bring valuable insight into the mechanism by which COVID-19 infection influences the hematological and biochemical parameters of patients with acute leukemia.

**Methods:**

This retrospective investigation uses repeated measures to examine changes in hematological and biochemical parameters among patients with acute leukemia before and after COVID-19 infection at a major Saudi tertiary center. The investigation was conducted at the Ministry of National Guard-Health Affairs in Riyadh, Saudi Arabia, on 24 acute leukemia patients with COVID-19 between April 2020 and July 2023. The impact of COVID-19 on clinical parameters, comorbidities, and laboratory values was evaluated using data obtained from the electronic health records at four designated time intervals. The relative importance of comorbidities, testing preferences, and significant predictors of survival was ascertained.

**Results:**

The majority of leukemic COVID-19-infected patients, primarily detected through PCR tests, were diagnosed with acute lymphoblastic leukemia (70.8%). The hematological and biochemical parameters exhibited stability, except for a brief increase in ALT and a sustained rise in AST. These changes were not statistically significant, and parameters remained normal at all time points. Additionally, an increase in monocyte count was shown at time point-3, as well as platelet counts at time point 2.

**Conclusion:**

While this study did not detect statistically significant effects of COVID-19 on biochemical and hematological parameters in acute leukemia patients, further investigation is needed to fully understand the potential adverse reactions and modifications following COVID-19 infection.

## Background

Coronaviruses (family Coronaviridae) are viruses with genomes comprising a single-stranded positive-sense RNA [1]. Following the severe acute respiratory syndrome (SARS) and Middle East respiratory syndrome (MERS) epidemics, which resulted in roughly 700 and 400 deaths, respectively, coronaviruses received significant scientific attention in the early 2000s [2]. Early in December, in and around Wuhan, China, the reporting of severe acute respiratory syndrome coronavirus 2 (SARS-CoV-2) concerned scientific communities about the disease known as coronavirus disease 2019 (COVID-19) [3, 4]. COVID-19 patients have severe respiratory abnormalities and breathing difficulties, which may eventually cause death [5]. The major factors contributing to the virus’s widespread distribution worldwide include its extremely infectious mode of transmission, its prolonged stability in the air, and inert surfaces like steel [6]. SARS-CoV-2 can infect host cells and proliferate despite having a single-strand positive-sense RNA genome and low structural and functional protein resources [7]. SARS-CoV-2 manipulates the host’s molecular machinery to complete its life cycle and create functioning virion progeny [8].

Awareness of the COVID-19 process is limited but expanding among cancer patients, particularly those with hematologic malignancies. Cancer patients may have a greater infection rate than the overall population [9, 10]. Only 10 of 1099 and 18 of 1590 COVID-19 patients in two Chinese investigations were diagnosed with cancer [11, 12]. In one trial, 60% of COVID-19-treated patients with blood malignancy recovered after a 14-day observation period [13]. Patients with leukemia are usually myelosuppressed, immunocompromised, and probably immunoglobulin deficient, rendering them more sensitive to COVID-19 [14]. Patients with leukemia may be at a much higher risk of getting SARS-CoV-2 infection due to the disease biology of leukemia subtypes, associated therapy, underlying comorbidities, patient-specific features, and unique COVID-19-related risk factors [14].

Undesirably, minimal hematological investigation was performed on COVID-19 infected leukemic patients [15]. Since COVID-19 is a recently emerged virus, it is uncertain whether variations occur in relation to other blood malignancies and how the virus impacts individuals with leukemia. Patients with blood cancer are vulnerable to SARS-CoV-2 infection due to immunocompromise caused by both disease and cancer treatment. Although the SARS-CoV-2 is well known for causing mild to severe pulmonary symptoms, it can also cause various extrapulmonary symptoms, including hematologic abnormalities [16, 17]. A dysfunctional immune response to viral infections can cause secondary mutational events that enhance clinical leukemia development [18]. Furthermore, SARS-CoV-2 has been shown to interact strongly with the renin-angiotensin system (RAS), which has been implicated in neoplastic hematopoiesis [19, 20].

The present study aims to explore the changes in the hematological and biochemical parameters in patients with acute leukemic before, during, and following COVID-19 infection in a repeated measure manner at a Major Saudi Tertiary Center. This report will give insight into the mechanism behind altering the hematological and biochemical parameters in patients with acute leukemia infected with COVID-19.

## Methods

### Study design and data collection

The ethical approval for the study was obtained from the institutional review board at King Abdullah International Medical Research Centre (KAIMRC) under the approval number (NRC23R/458/08). Following ethical approval, the research team members contacted the medical records unit, research department, and data management section at the KAIMRC in the Ministry of National Guard-Health Affairs, Riyadh, Saudi Arabia, for data collection. The information was obtained from the patient’s electronic health records using hospital data management systems. Twenty-four acute leukemia patients who tested positive for COVID-19 via polymerase chain reaction or rapid antigen testing between the beginning of April 2020 and the end of July 2023 were recruited for this study. Because this is a retrospective study, the KAIMRC ethics committee/institutional review board waived the requirement for informed consent, and all identifying information was removed to protect patients’ confidentiality. The study was conducted in accordance with the Helsinki Declaration and local institutional standards.

In an attempt to assess the impact of COVID-19 infection on acute leukemia patients, four-time points were specified. These include pre-COVID-19 time-point (Time 0; 1–3 months before COVID-19 infection), during COVID-19 infection time-point (Time 1; within 6 days of COVID-19 infection), first post-COVID-19 time-point (Time 2; 1–3 months after COVID-19 infection), and second post-COVID-19 time-point (Time 3; 4–6 months after COVID-19 infection).

Clinical data includes demographic data and comorbidities such as hypertension, coronary artery disease, arrhythmia, diabetes, Chronic Obstructive Pulmonary Disease (COPD), asthma, end-stage renal disease (ESRD), *Chronic kidney disease* (*CKD*), hypogammaglobulinemia, *hematopoietic stem cell transplant* (SCT) and Peripheral Blood SCT. In addition, laboratory data consisted of biochemical and hematological parameters. The biochemical profile includes alanine transaminase (*ALT*), aspartate aminotransferase (*AST*), creatinine, blood Urea Nitrogen (BUN), blood sugar Test, estimated glomerular filtration rate (*eGFR)*, chloride, potassium, and sodium. Hematological parameters include neutrophils, eosinophils, basophils, lymphocytes, monocytes, platelet count (PLT), and red blood cell count (RBC).

The Exclusion Criteria include: Patients with other concurrent malignancies, Patients with a history of bone marrow transplantation. Also, Patients with significant comorbidities affecting hematological or biochemical parameters independent of leukemia or COVID-19, Patients with incomplete medical records or missing data necessary for analysis. Further, Patients with a history of prior COVID-19 infection before the onset of leukemia, Patients with active infections other than COVID-19, Patients with a history of significant hematological disorders unrelated to leukemia.

### Statistical analysis

Statistical analysis was performed using SPSS® software version 25.00 (IBM Corp., Armonk, NY, USA), and the graphical presentation was carried out using GraphPad Prism version 9.4.1 (GraphPad Software Inc., San Diego, CA, USA). Descriptive data were presented as medians and interquartile ranges (IQR) for continuous data, whereas percentages and frequencies were used for categorical data. A one-way repeated measures ANOVA (also known as a within-subjects ANOVA) was performed to assess changes in the laboratory parameters between post-COVID-19 time points and pre-COVID-19 time points or during COVID-19 time-point. A p-value < 0.05 was considered significant.

## Results

### Sample characteristics

Table 1 presents a comprehensive overview of the demographic and clinical characteristics of the studied population, shedding light on the distribution of key variables and contributing factors within the cohort. Among the individuals, 45.8% were female, with a median age of 19 years (IQR; 23.4–41.3). The primary diagnoses varied, with the majority (70.8%) suffering from acute lymphoblastic leukemia, followed by 25% with acute myeloid leukemia and 4.2% with acute promyelocytic leukemia. A subset of the population had comorbidities, with 16.7% experiencing hypertension, 8.3% with diabetes, and 16.7% with asthma. Notably, none of the individuals had coronary artery disease, arrhythmia, chronic obstructive pulmonary disease (COPD), end-stage renal disease (ESRD), or hypogammaglobulinemia. Additionally, 4.2% had chronic kidney disease (CKD), and a similar percentage had undergone hematopoietic stem cell transplant (SCT). Peripheral blood SCT was reported in 29.2% of cases. The study observed a mortality rate of 12.5%. Regarding COVID-19 testing, 83.3% underwent PCR testing, while 16.7% opted for rapid antigen testing.

**Table 1:** Characteristics of patients included in the study

Characteristics	Descriptive Statistics
**Female sex**, n (%)	11 (45.8)
**Age**, median (IQR), y	19 (23.4–41.3)
**Primary Diagnosis**, n (%)	
Acute lymphoblastic leukemia	17 (70.8)
Acute myeloid leukemia	6 (25)
Acute promyelocytic leukemia	1 (4.2)
**Hypertension**, n (%)	4 (16.7)
**Diabetes**, n (%)	2 (8.3)
**Asthma**, n (%)	4 (16.7)
**CKD**, n (%)	1 (4.2)
**Hypogammaglobulinemia**, n (%)	0 (0)
**Hepatopoietic SCT**, n (%)	1 (4.2)
**Peripheral Blood SCT**, n (%)	7 (29.2)
**Mortality**, n (%)	3 (12.5)
**Type of Covid exam**, n (%)	
COVID-19 PCR	20 (83.3)
Rapid Antigen Testing	4 (16.7)
**Year of the infection**, n (%)	
2020	1 (4.2)
2021	15 (52.5)
2022	8 (33.3)
2023	0 (0)

COPD; Chronic Obstructive Pulmonary Disease, ESRD; End-Stage Renal Disease, CKD; Chronic Kidney Disease, SCT; Stem Cell Transplantation

### Biochemical and hematological parameters

**Fig. 1:**
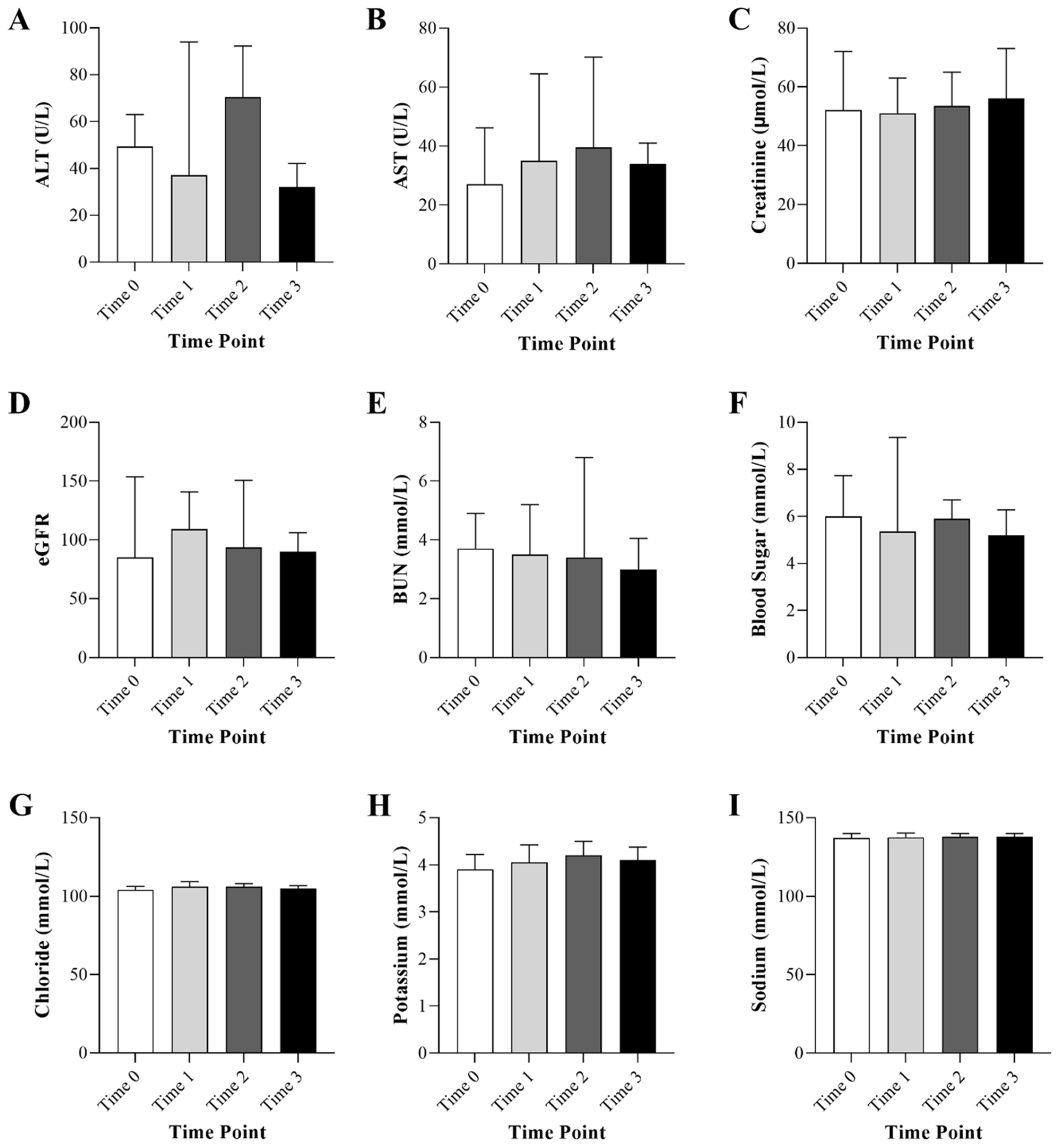
Biochemical parameters of acute leukemia patients included in the study at different time points of pre-COVID-19 time point (Time 0; 1–3 months before COVID-19 infection), during COVID-19 infection (Time 1; with 6 days of COVID-19 infection), first post-COVID-19 time point (Time 2; 1–3 months after COVID-19 infection), and second post-COVID-19 time point (Time 3; 4–6 months after COVID-19 infection). (**A**) Alanine Transferase (ALT), (**B**) Aspartate Aminotransferase (AST), (**C**) Creatinine, (**D**) Estimated glomerular filtration rate (eGFR), (**E**) Urea Nitrogen (BUN), (**F**) Blood Sugar, (**G**) Chloride, (**H**) Potassium, and (**I**) Sodium

Figure 1 presents the biochemical parameters of subjects included in this investigation at the study time points. No significant differences were found between time points. Of note, the median of ALT was above the normal range during the first post infection time-point (37.0 U/L, IQR; 17.5–94.0), which returned to the normal level at the second post-COVID-19 time-point (19.0 U/L, IQR; 13.5–35.5). Moreover, the level of AST was higher than the normal range at all time points during and after SARS-CoV-2 infection. The remaining parameters were generally within the normal ranges at all-time points.

**Fig. 2:**
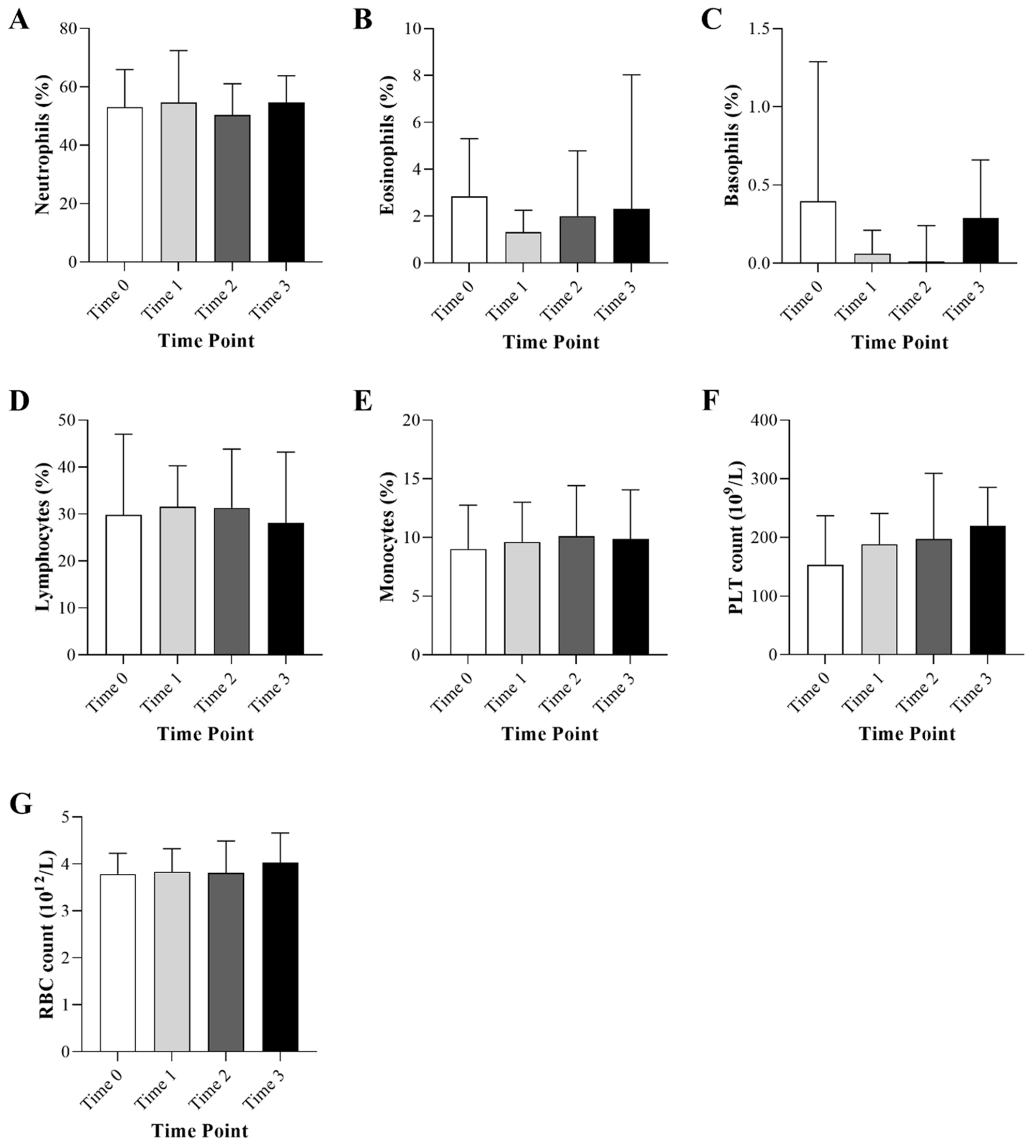
Hematological parameters of acute leukemia patients included in the study at different time points of pre-COVID-19 time point (Time 0; 1–3 months before COVID-19 infection), during COVID-19 infection (Time 1; with 6 days of COVID-19 infection), first post-COVID-19 time point (Time 2; 1–3 months after COVID-19 infection), and second post-COVID-19 time point (Time 3; 4–6 months after COVID-19 infection). (**A**) Neutrophils, (**B**) Eosinophils, (**C**) Basophils, (**D**) Lymphocytes, (**E**) Monocytes, (**F**) Platelet count (PLT), and (**G**) Red Blood Cell Count (RBC)

Figure 2 shows an overview of the hematological parameters at the study time points. No statistically significant differences between time points were observed. However, monocyte count showed a marginal increase at the post-COVID-19 time-point (time 3) (10.1%, IQR; 5.9–14.4) compared to other time points (9.0%, IQR 8.1–12.8 at Time 0, 9.6%, IQR; 5.8–13.0 at Time 1, and 9.9%, IQR; 7.5–14.1 at Time 3) (*p* = 0.05). Although statistically significant were not achieved, platelet counts showed a substantial increase at post-COVID-19 time points (197 10^9^/L, IQR; 109-309.3 at Time 2 and 220 10^9^/L, IQR; 119–285 at Time 3) compared to pre-COVID-19 and during COVID-19 time points (153 10^9^/L, IQR; 76.5-236.8 at Time 0 and 188.5 10^9^/L, IQR; 104-240.8 at Time 1) (*p* = 0.07). Furthermore, RBC count showed a marginal decrease during COVID-19 infection (3.8 10^12^/L, IQR; 2.9–4.3) compared to the second post-COVID-19 time-point (4.03 10^12^/L, IQR; 3.1–4.7) (*p* = 0.05).

## Discussion

Since the available data on acute leukemia patients with COVID-19 is limited to small patient cohorts, case reports/series, or expert comments, there is a knowledge gap in this area [24]. Because of this gap, determining the most effective care plan for acute leukemia patients during the COVID-19 pandemic has proven challenging [21, 22]. Therefore, this study was undertaken to evaluate the effect of COVID-19 on the hematological and biochemical parameters of acute leukemia patients in four-time points; Pre-COVID-19 (Time 0; 1–3 months before COVID-19 infection), COVID-19 infection (Time 1; within 6 days of COVID-19 infection), COVID-19 infection (Time 2; 1–3 months following COVID-19 infection), and COVID-19 infection (Time 3; 4–6 months following COVID-19 infection).

The demographic profile of the studied subjects illustrates a relatively equitable gender distribution, as 45.8% of the participants were female. The median age of the studied population was 19 years, suggesting a considerable interquartile range (IQR) of 23.4–41.3, further underscoring the relatively younger ages of studied subjects. A noteworthy characteristic of this study is the distribution of primary diagnoses; acute lymphoblastic leukemia afflicts 70.8% of the participants, followed by acute myeloid leukemia at 25% and acute promyelocytic leukemia at 4.2%. The analysis presents significant findings regarding the frequency of various types of leukemia among the participants, laying the groundwork for additional research into the ramifications of these particular diagnoses. In relation to COVID-19 testing, PCR testing was performed on most individuals (83.3%), underscoring the criticality of precise diagnostic techniques.

Comorbidity identification is essential in comprehending the overall health condition of the studied population. It is worth mentioning that this cohort did not have any individuals with heart disease, arrhythmia, chronic obstructive pulmonary disease (COPD), end-stage renal disease (ESRD), or hypogammaglobulinemia. This cohort did have comorbidities such as hypertension (16.7%), diabetes (8.3%), and asthma (16.7%), which highlights the criticality of taking into account these elements. Significantly high rates of chronic kidney disease (CKD) and hematopoietic stem cell transplant (SCT) (4.2% each) suggest that the study population possesses additional complexities. Furthermore, the notable occurrence of peripheral blood SCT in 29.2% of instances underscores the importance of evaluating various stem cell transplant modalities within the clinical setting.

A study performed by Mato et al. involved 198 subjects with chronic lymphocytic leukemia (CLL) from 43 international centers with a median age of 70.5 years [23]. The death rate of the studied subjects was 33%. That study suggested that hospitalized CLL patients with COVID-19 are at high risk of death regardless of their age, disease phase, or treatment status. However, this conclusion was contradicted by another study involving 90 patients with CLL. The study suggested that although more severe COVID-19 complications were correlated with age, no significant association was obtained between advanced age in CLL patients infected with SARS-COV-2 and the death rate [24].

The selection bias and methodological variances could be the cause of these disparities. The relatively low mortality rate (12.5%) we observed in this study further supports the conclusion that there is no association between age and fatality rate resulting from COVID-19 in acute leukemia patients. However, this finding needs further confirmation by other studies as the median age of the studied participants in this report was 19 (IQR) of 23.4–41.3, which does not encompass a wide range of ages. Also, because patients in this study may have been older or less fit when their condition started, these findings should be read with caution [25].

The lack of substantial variations among time points studied in our report offers valuable insights into the stability of multiple parameters being examined. The results of this study showed that, on average, the measured variables did not undergo significant fluctuations throughout the studied time points. It is imperative to comprehend the temporal consistency of these parameters in order to decipher the study’s wider implications and trends. Interestingly, we observed an increase in ALT levels of the studied subjects in SARS-CoV-2 post-infection (Time-point 2) before the restoration of baseline levels. In addition, the results of this study showed a persistent increase in AST levels at each time point post-infection. These findings are consistent with previous studies indicating that COVID-19 causes elevated liver enzyme activities in patients with or without chronic liver diseases [24, 26, 27].

The increase in the ALT and AST levels could be mediated via direct or indirect mechanisms. For instance, while the SARS-CoV-2 primary receptors, angiotensin-converting enzyme 2 (ACE-2), are mainly found in the lungs, they are also highly expressed in other organs, including the liver [28]. Therefore, SARS-CoV-2 can attach and replicate in the hepatocytes, causing direct damage to the infected cells and releasing ALT and AST in the bloodstream [30]. Furthermore, it has been reported that SARS-CoV-2 infection results in excessive cytokine production (Cytokines storm), especially in severe cases, which eventually causes damage to the body organs, such as the liver, and hence increases liver enzyme activities [29, 30]. The investigation of hematological parameters at multiple time intervals in this study offers more information regarding the possible effects of COVID-19 on the blood profile. Although no significant statistical differences were obtained in most hematological parameters across the study time points, it is important to consider specific trends and marginal changes.

A noteworthy observation is the elevation in the proportion of monocytes observed at time point two following the onset of COVID-19 compared to the remaining time intervals. This finding is consistent with a previous report by Park et al., which showed a significant elevation of monocytes beyond the acute COVID-19 infection [31]. Monocytes are known as important cellular regulators of COVID-19 pathogenesis. It has been shown that monocytes undergo dysregulation during acute SARS-CoV-2 infection, displaying abnormal functions and contributing to the cytokine storm observed in severe cases of COVID-19 [32]. Furthermore, a study involving 32 participants with confirmed COVID-19 tests showed overexpression of CD169, a type I interferon-inducible receptor on monocytes, in 93.7% of the studied subjects [33]. In addition, the increase in the expression levels of CD169 has been reported in various health conditions, including viral infections [34]. The observed increase in the proportion of monocytes in this study may indicate a distinct inflammatory or immune response following COVID-19 infection. Although the findings of this study didn’t show a significant increase in monocytes following COVID-19 in leukemic patients, the results encourage additional research to study the immune dynamics that occur during the recovery phase following COVID-19 in this population.

Studies assessing the impact of COVID-19 on platelet count yield contradicting findings. Thrombocytopenia has been reported following several viral infections such as hepatitis B virus, hepatitis C virus, cytomegalovirus (CMV), human immunodeficiency virus (HIV), and Zika virus [35]. In addition, a decrease in platelet count was correlated with the disease’s severity [36]. On the other hand, Shiyu et al. reported that COVID-19 patients had higher platelet counts than their healthy counterparts [37]. The study suggested the increase in platelet count could be attributed to the reactive increase of thrombopoietin following pulmonary inflammation. Furthermore, multiple studies showed that platelet peak was higher in severe COVID-19 cases compared to mild and moderate cases [38, 39]. One of the interesting findings of our study is the substantial increase in platelet count observed at times 2 and 3 post-COVID-19 compared to the time points 0 and 1. Platelets play critical roles in clotting formation and immune response. It is well-known that platelets are involved in the immune response by secreting various cytokines and chemokines to regulate inflammatory functions such as leucocyte migration, phagocytosis, and reactive oxygen species (ROS) generation [40].

Our findings suggest the increase in platelet counts may be a marker of respiratory tract inflammatory reactions resulting from SARS-CoV-2 infection in the studied subjects. Although the p-value is 0.07, marginally below the conventional threshold for significance, this pattern merits consideration in comprehending the hematological ramifications of COVID-19 in leukemic patients. Multiple studies showed different alterations in hematological parameters in COVID-19 patients, such as elevated ferritin levels and reduced RBC count and hemoglobin levels. These changes were reported to be associated with the disease progression and severity [41, 42].

Our study showed a slight reduction in RBC count observed during the initial time point following COVID-19 infection (time 1) compared to time point 3, indicating possible temporary impacts on erythropoiesis throughout the acute phase of the infection. The effect of SARS-CoV-2 infection on RBC reduction could be attributed to the direct infection of RBC precursors by the virus, which results in a reduction in erythrocyte turnover and low Hb levels [43]. In addition, a study performed by Mullen et al. showed COVID-19 patients had higher levels of reactive oxygen species, which can damage RBCs and reduce their oxygen-carrying capacity [44].

Recent research has shown diverse patient groups with co-occurring COVID-19 infection and a range of hematological disorders. The literature has details regarding the course of the disease, mortality, and treatment results for acute leukemia patients with COVID-19 [45]. Ferrara and others revealed that ten acute myeloid leukemia (AML) patients with COVID-19 had a 50% death rate [46]. Several studies demonstrated that patients with hematological malignancies are more likely to contract COVID-19 [47] and that cancer patients are more susceptible to poorer outcomes and may experience more serious complications than those who do not have cancer and COVID-19 [12].

The largest investigation of COVID-19 infection in hematological malignancies was conducted by Passamonti et al., who examined 536 cases of hematological cancer infected with SARS-CoV-2 with symptoms. 51 patients (10%) with AML and 16 patients (3%) with Acute lymphoblastic leukemia (ALL) were included in that cohort. Out of the 536 patients, 178 (or 37%) did not survive; AML and ALL were the two most common causes of death in the non-survivor group. According to their report, patients who suffered from COVID-19 and hematological malignancies were at a higher risk of dying. Poorer outcomes in their cohort have been linked to advanced age, progressive disease, AML, non-Hodgkin’s lymphoma, and plasma cell neoplasms [48]. Moreover, Martin-Moro and colleagues revealed the outcomes of 34 cases of hematological malignancies, seven of which were leukemia cases, and the death rate for patients with COVID-19 infection was found to be 36% [49]. Due to their compromised immune systems, leukemia patients often experience several complications during their treatment, including severe infections brought on by leukopenia or neutropenia [50].

Our study is limited by the need for more information on the stage of leukemia and COVID-19 and the type of treatment among the groups for which clinical data were collected. Such information is critical for ensuring that the study’s findings are relevant and applicable to patient care and treatment methods and for understanding the disease’s influence on hematological and biochemical markers at different stages of disease and recovery. In addition, it is noted that the sample size of 24 patients with acute leukemia may be considered small. However, due to the retrospective study nature, the available sample size is frequently limited by factors such as the rarity of the ailment being investigated and the availability of thorough patient data. Although the small sample size, this study is important because it advances our understanding of the relationship between COVID-19 and acute leukemia, specifically concerning hematological and biochemical factors.

## Conclusions

This study uses a four-time point assessment to understand better how COVID-19 affects acute leukemia patients. The biochemical parameters were steady before and after SARS-CoV-2 infection in the leukemic patients except for a temporary ALT increase and a protracted AST increase. Hematological results showed elevations in monocytes and platelets count post-infection, indicating their potential inflammatory roles due to the viral infection. The outcomes of COVID-19 in leukemic patients in this study were similar to those in the literature. Selection bias and small sample size are the main limitations of this study, and further investigation is needed in this area.

sample size are the main limitations of this study, and further investigation is needed in this area.

## Data Availability

Might be provided upon request to the corresponding author. The datasets used and/or analyzed during the current study are available from the corresponding author on reasonable request.

## Abbreviations

COVID-19: Coronavirus disease 2019
RNA: Ribonucleic acid
PCR: Polymerase chain reaction
SARS: Severe acute respiratory syndrome
MERS: Middle East respiratory syndrome
SARS-CoV-2: Severe acute respiratory syndrome coronavirus 2
RAS: Renin-angiotensin system
KAIMRC: King Abdullah International Medical Research Centre
COPD: Chronic Obstructive Pulmonary Disease
ESRD: End-stage renal disease
CKD: Chronic kidney disease
SCT: Stem cell transplant
ALT: Alanine transaminase
AST: Aspartate aminotransferase
BUN: Blood Urea Nitrogen
eGFR: Estimated glomerular filtration rate
PLT: Platelet count
RBC: Red blood cell count
IQR: Interquartile ranges
CLL: Chronic lymphocytic leukemia
ACE-2: Angiotensin-converting enzyme 2
CMV: Cytomegalovirus
ROS: Reactive oxygen species
AML: Acute myeloid leukemia
ALL: Acute lymphoblastic leukemia
